# MEMS Gyroscope Automatic Real-Time Mode-Matching Method Based on Phase-Shifted 45° Additional Force Demodulation

**DOI:** 10.3390/s18093001

**Published:** 2018-09-07

**Authors:** Feng Bu, Dacheng Xu, Heming Zhao, Bo Fan, Mengmeng Cheng

**Affiliations:** School of Electronic and Information Engineering, Soochow University, Suzhou 215006, China; bf_suda@126.com (F.B.); xudacheng@suda.edu.cn (D.X.); 20164028006@stu.suda.edu.cn (B.F.); 20164228010@stu.suda.edu.cn (M.C.)

**Keywords:** MEMS gyroscope, real-time mode-matching, 45° additional force, phase-shifted demodulation, negative stiffness effect

## Abstract

In order to solve the problem where existing mode-matching methods in microelectromechanical systems (MEMS) vibrating gyroscopes fail to meet real-time and reliability requirements, this paper presents a novel method to accomplish automatic and real-time mode-matching based on phase-shifted 45° additional force demodulation (45° AFD-RM). The phase-shifted 45° additional force signal has the same frequency as the quadrature force signal, but it is phase-shifted by 45° and applied to the sense mode. In addition, two-way phase-shifted 45° demodulations are used at the sense-mode detection output to obtain a phase metric that is independent of the Coriolis force and can reflect the mode-matching state. Then, this phase metric is used as a control variable to adaptively control the tuning voltage, so as to change the sense-mode frequency through the negative stiffness effect and ultimately achieve real-time mode-matching. Simulation and experimental results show that the proposed 45° AFD-RM method can achieve real-time matching. The mode frequency split is controlled within 0.1 Hz, and the gyroscope scale factor, zero-bias instability, and angle random walk are effectively improved.

## 1. Introduction

Microelectromechanical systems (MEMS) vibratory gyroscopes have the advantages of small size, light weight, and low cost [1], resulting in wide application prospects in military and civilian fields. The oscillation amplitude greatly increases when the oscillator vibrates at the resonant frequency. Therefore, the mechanical sensitivity is maximized and the signal-to-noise ratio can be effectively improved when the two modes of the gyroscope have the same resonant frequency (mode-matched) [2,3]. However, because of fabrication imperfections, it is difficult to completely match the resonant frequencies of the two modes through structural design [4]. Therefore, mode-matching through electrostatic tuning is often adopted; this has become a research focus for MEMS gyroscopes.

At present, the popular tuning method is to use the negative-stiffness electrostatic effect of the flat-plate capacitor structure and apply DC voltage on the flat-plate electrode to change the mode frequency to achieve mode matching [5,6,7]. The mode-matching method is divided into one-time matching and real-time matching. One-time matching is achieved through manual adjustment or one-time automatic matching. Manual adjustment is performed by sweeping at different tuning voltages to determine tuning voltage values, but this process is time-consuming and has poor stability. One-time automatic matching is mainly based on the frequency characteristics (phase-frequency and amplitude-frequency) of the gyroscope vibration mode. For example, the phase-frequency characteristic is that the phase delay between the quadrature input and output of the sense mode is −90° when mode-matched. This characteristic was used to achieve mode matching in [8,9,10], and the phase-locked loop (PLL) technique was used to adjust the tuning voltage. The amplitude-frequency characteristic is the quadrature response signal amplitude being maximized when mode-matched. This characteristic was used in [11,12]. However, the signal out of the Coriolis demodulation channel is used to control the frequency tuning voltage in these methods. As a result, only when the tuning voltage is fixed and the matching loop is cut off after mode-matching can the angular rate be measured. For this reason, these methods cannot achieve real-time mode-matching. However, in practical applications, the frequency split of the gyroscope mode varies with changes in environmental parameters [13,14,15]. Therefore, one-time matching cannot meet the requirements.

Therefore, a real-time mode-matching method that does not affect normal angular rate detection is urgently needed. At present, there are only a few studies on real-time matching, which can be divided mainly into the external force method and the system compensation method. The external force method applies an external load on the sense mode and detects its response information to achieve mode-matching. For example, a low-frequency disturbance signal is additionally applied to the sense-mode in [16], and real-time matching is achieved by detecting the maximum amplitude of the vibration caused by the disturbance signal. However, the accuracy of the maximal value search is not high, and the disturbance signal is not completely eliminated from the Coriolis detection loop, which affects the normal Coriolis demodulation output. An external force signal in phase with the Coriolis force is applied to the sense mode in [17], which achieved real-time matching by detecting the phase information of the external force signal. However, it requires the response amplitude of the external force to be much larger than that of the Coriolis and quadrature force in order to achieve accurate matching, which limits its application. The system compensation method adaptively adjusts the sense-mode frequency according to environmental changes by an intelligent system. For example, a fuzzy neural network is used to adaptively adjust the tuning voltage to achieve real-time matching in [18]. However, this method needs to learn the frequency split data in different environments for each gyroscope in advance, which is difficult to achieve in practical applications. Thus, existing real-time mode-matching methods have problems such as influencing angular rate detection and difficulty in implementation.

This paper proposes a new real-time mode-matching method called phase-shifting 45° additional force demodulation (45° AFD-RM). By applying a phase-shifted 45° additional force signal on the sense mode to obtain a phase metric and using this phase metric to adjust the tuning voltage through a proportional-integral (PI) controller, real-time mode-matching is finally realized. Section 2 introduces the gyroscope dynamic model and system characteristics in mode-matching. Section 3 describes the design framework of the 45° AFD-RM mode-matching method. Section 4 and Section 5 provide simulation and experimental results, respectively, showing the benefits of 45° AFD-RM, mainly in scale factor and zero-bias stability.

## 2. Gyroscope Model

### 2.1. Gyroscope Dynamic Model

**Figure 1:**
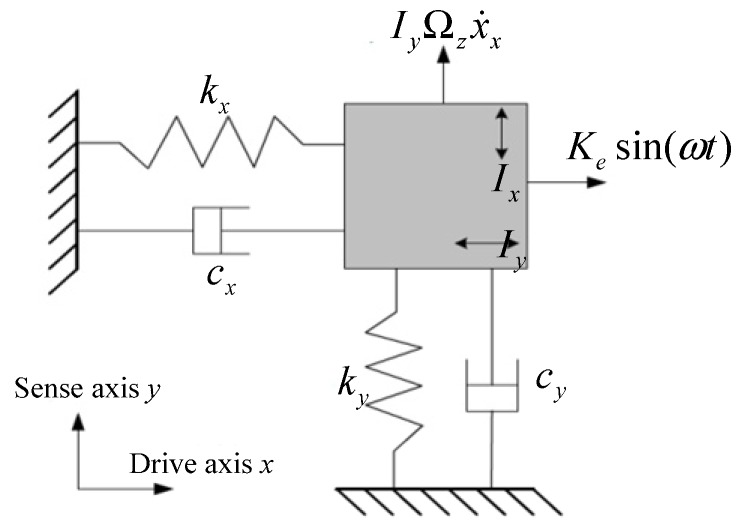
Gyroscope dynamic model.

The vibratory gyroscope is composed of a drive mode and sense mode. The mode dynamic model can be described by a second-order mass-damper-spring system [19], as shown in Figure 1. The gyroscope dynamic equation is

(1) $$\left\{ \begin{array}{l} m\ddot{x}+c_{x}\dot{x}+k_{x}x=F(t)\\ m\ddot{y}+c_{y}\dot{y}+k_{y}y=-2m\Omega \dot{x}-k_{yx}x \end{array}\right.$$

where x and y are the vibration displacement in drive mode (x-axis) and sense mode (y-axis), m is the mode mass, F(t) is the driving force (i.e., $F(t)=\cos(\omega_{d}t)$), c and k are the damping and stiffness coefficients, Ω is the angular rate input, $2m\Omega \dot{x}$ is the Coriolis force, $k_{yx}\;x$ is the stiffness coupling force, and $k_{yx}$ is the stiffness coupling coefficient from the sense mode to the drive mode.

For the drive mode, the transfer function is given as

(2) $$H\left(s\right)=\frac{X\left(s\right)}{F\left(s\right)}=\frac{1/m}{s^{2}+\frac{\omega_{x}}{Q_{x}}s+\omega_{x}^{2}}$$

where $\omega_{x}$ and $Q_{x}$ are the natural resonant frequency and quality factor (Q) of the drive mode, respectively. The displacement and phase expressions of the drive mode during steady state are:

(3) $$\left\{ \begin{array}{l} x\left(t\right)=A_{x}\cos\left(\omega_{d}t+\varphi_{x}\right)\\ \varphi_{x}=\arctan\frac{\omega_{d}\omega_{x}}{\left(\omega_{d}^{2}-\omega_{x}^{2}\right)Q_{x}} \end{array}\right.$$

where $\omega_{d}$ is the drive signal frequency. From these equations, the phase delay $\varphi_{x}=-90^{{}^{\circ}}$ when $\omega_{d}=\omega_{x}$.

For the sense mode, its transfer function, displacement, and phase characteristics are consistent with those of the drive mode. However, its inputs are the stiffness coupling force (quadrature force) and the Coriolis force. The Coriolis force is proportional to the vibration speed $\dot{x}(t)$ of the drive mode, i.e., $F_{\Omega }(t)=-2m\Omega (t)\dot{x}(t)$, in which Ω(t) is the angular rate, i.e., $\Omega \left(t\right)=\Omega \cos\left(\omega_{\Omega }t\right)$. The quadrature force is proportional to the vibration displacement x(t) of the drive mode, i.e., $F_{q}(t)=-k_{yx}x(t)$.

The sense-mode displacement $y_{q}(t)$ and $y_{\Omega }(t)$ caused by the quadrature force and the Coriolis force are:

(4) $$\left\{ \begin{array}{l} y_{q}(t)=-A_{ys}k_{yx}A_{x}\sin(\omega_{d}t+\varphi_{y})\\ y_{\Omega }(t)=-A_{ys}m_{y}\omega_{d}\Omega \left(t\right)\cos(\omega_{d}t+\varphi_{y}) \end{array}\right.$$

(5) $$\left\{ \begin{array}{l} A_{ys}=\frac{1}{\sqrt{{\left(\omega_{y}^{2}-\omega_{x}^{2}\right)}^{2}+{\left(\frac{\omega_{y}\omega_{x}}{Q_{y}}\right)}^{2}}}\\ \varphi_{y}=-\arctan\frac{\omega_{y}\omega_{x}}{Q_{y}\left(\omega_{y}^{2}-\omega_{x}^{2}\right)} \end{array}\right.$$

where $\varphi_{y}$ is the phase delay caused by the sense mode. When the resonant frequencies of the two modes are equal ($\omega_{y}=\omega_{x}$), then $\varphi_{y}=-90^{{}^{\circ}}$. This means that this phase delay information can be used to determine whether the two modes are matched.

### 2.2. System Characteristics in Mode-Matching

**Figure 2:**
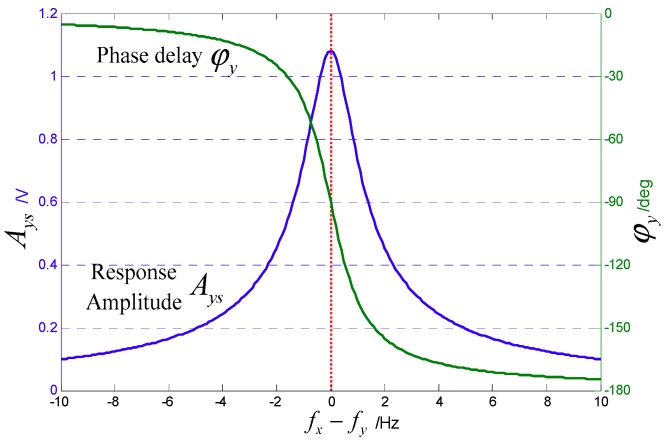
Amplitude frequency and phase frequency characteristics of sense mode.

Because of fabrication imperfections, there is a frequency split between the two modes of the gyroscope. According to Equation (5), the vibration response and phase characteristics at different mode frequency splits ($f_{x}-f_{y}$) are shown in Figure 2, where the sense-mode $Q_{y}$ is set to 5000.

It can be seen that when the two mode frequencies are equal, the vibratory response of the sense mode is maximized and the phase delay $\varphi_{y}=-90^{{}^{\circ}}$. For the Coriolis response, the gyroscope mechanical sensitivity $S_{m}$ is given as

(6) $$S_{m}=\left|\frac{y_{\Omega }}{\Omega }\right|=\frac{2A_{x}\omega_{d}}{\sqrt{{\left(\omega_{y}^{2}-\omega_{x}^{2}\right)}^{2}+{\left(\frac{\omega_{y}\omega_{x}}{Q_{y}}\right)}^{2}}}$$

When $\omega_{y}=\omega_{x}$, the sensitivity also reaches its maximum. For this reason, mode-matching is highly important to improve the mechanical sensitivity of MEMS gyroscopes and the stability of the zero bias [20].

### 2.3. Electrostatic Negative Stiffness Tuning

Mode-matching through electrostatic tuning is usually based on the electrostatic negative stiffness effect [21]. In a gyroscope structure, a set of electrodes with a flat capacitance structure is usually designed as a tuning electrode in sense mode. When DC voltage is applied to the tuning plate electrode, the equivalent stiffness k of the oscillator exhibits a negative quadratic correlation with the tuning voltage $V_{p}$; also, increasing the voltage reduces the equivalent stiffness, thereby reducing the sense-mode resonance frequency.

With S as the area of the flat-plate capacitor, $d_{0}$ the distance between the plates, d the displacement of the moving plate, ε the vacuum dielectric constant, $V_{p}$ the tuning voltage, and $\omega_{0}^{}$ the initial resonant frequency, the relationship between the tuning voltage $V_{p}$ and resonant frequency ω is:

(7) $$\omega =\sqrt{\omega_{0}^{2}-bV_{p}{}^{2}},\;b=\frac{\varepsilon S}{m{\left(d_{0}-d\right)}^{3}}$$

## 3. 45° AFD-RM Mode-Matching Method

### 3.1. Method Framework

In drive mode, the drive signal is $\cos(\omega_{d}t)$ and uses traditional phase-locked loop and automatic gain control (PLL + AGC) closed-loop control to achieve drive-mode resonance ($\omega_{x}=\omega_{d}$). At steady state, the drive vibration displacement response is $x(t)=A_{x}\sin(\omega_{d}t)$. Thus, the Coriolis force is $F_{\Omega }(t)=-A_{x}m_{y}\omega_{d}\Omega \left(t\right)\cos(\omega_{d}t)$, and the quadrature force is $F_{q}(t)=-A_{x}k_{yx}\sin(\omega_{d}t)$.

**Figure 3:**
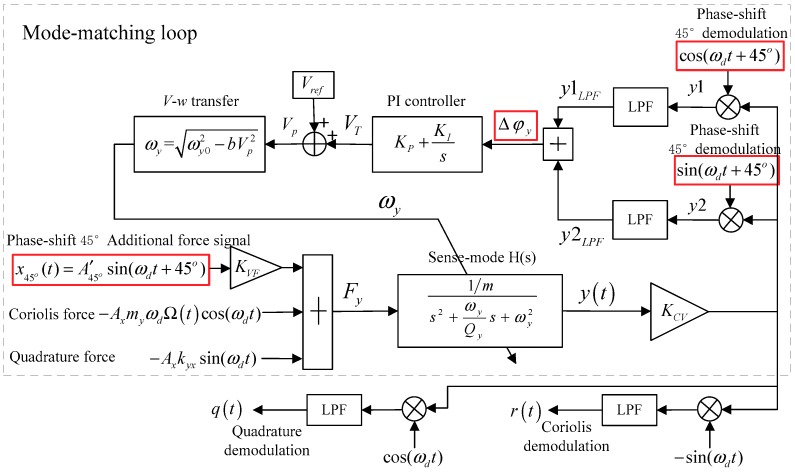
Control system framework of phase-shifting 45° additional force demodulation (45° AFD-RM).

A block diagram of the 45° AFD-RM method is shown in Figure 3. First, an additional force signal $x_{45^{{}^{\circ}}}(t)=A_{45^{{}^{\circ}}}^{\prime }\sin(\omega_{d}t+45^{{}^{\circ}})$ shifts the reference signal $\sin(\omega_{d}t)$ forward by 45°, and it works with the Coriolis force and quadrature force on the sense mode at the same time. Then, the phase-shift signal $\cos(\omega_{d}t+45^{{}^{\circ}})$ and $\sin(\omega_{d}t+45^{{}^{\circ}})$ demodulate the sense-mode output signal. After that, the two demodulated signals are passed through a low-pass filter (LPF) and added to obtain a phase metric $\Delta \varphi_{y}$ that can reflect whether the modes are matched. Finally, according to $\Delta \varphi_{y}$, the PI controller adjusts the tuning voltage $V_{T}$ to change the sense-mode frequency, and finally makes $\Delta \varphi_{y}=0$, achieving real-time mode-matching.

### 3.2. Mode-Matching Loop Analysis

The force generated by the phase-shifted 45° additional force signal on the sense mode is $F_{45^{{}^{\circ}}}(t)=x_{45^{{}^{\circ}}}(t)K_{VF}$. Then, the combined force input into the sense mode is $F_{y}(t)=F_{\Omega }(t)+F_{q}(t)+F_{45^{{}^{\circ}}}(t)$. Therefore, the vibration displacement response of the sense mode is

(8) $$\begin{array}{l} y(t)=\underset{\mathrm{Coriolis}\;\mathrm{response}}{\underset{︸}{\frac{A_{\Omega }}{2}\left(\cos\left((\omega_{d}+\omega_{\Omega })t+\varphi_{\omega_{d}+\omega_{\Omega }}\right)+\cos\left((\omega_{d}-\omega_{\Omega })t+\varphi_{\omega_{d}-\omega_{\Omega }}\right)\right)}}\\ +\underset{\mathrm{Quadrature}\;\mathrm{response}}{\underset{︸}{\;A_{q}\sin(\omega_{d}t+\varphi_{\omega_{d}})}}+\underset{45^{{}^{\circ}}\mathrm{additional}\;\mathrm{force}\;\mathrm{response}}{\underset{︸}{A_{45^{{}^{\circ}}}\sin(\omega_{d}t+45^{{}^{\circ}}+\varphi_{\omega_{d}})}} \end{array}$$

where the Coriolis response amplitude is $A_{\Omega }=-A_{x}m_{y}\omega_{d}\Omega A_{ys}$, the quadrature response amplitude is $A_{q}=-A_{x}k_{yx}A_{ys}$, and the additional force response amplitude is $A_{45^{{}^{\circ}}}=A_{45^{{}^{\circ}}}^{\prime }A_{ys}$. $A_{ys}$ is the sense-mode amplitude gain in the steady state, and $\varphi_{\omega_{d}}$ is the sense-mode phase delay when the input signal frequency is $\omega_{d}$.

Then, y(t) is demodulated by reference signals $\cos(\omega_{d}t+45^{{}^{\circ}})$ and $\sin(\omega_{d}t+45^{{}^{\circ}})$, and these two demodulated signals are filtered by LPF to obtain

(9) $$\begin{array}{l} y1_{LPF}=LPF\left\{y(t)\times \cos(\omega_{d}t+45^{{}^{\circ}})\right\}\\ \;=\frac{A_{\Omega }}{4}\left(\cos(\omega_{\Omega }t+\varphi_{\omega_{d}+\omega_{\Omega }}-45^{{}^{\circ}})+\cos(-\omega_{\Omega }t+\varphi_{\omega_{d}-\omega_{\Omega }}-45^{{}^{\circ}})\right)\\ +\frac{A_{q}}{2}\sin(\varphi_{\omega_{d}}-45^{{}^{\circ}})+\frac{A_{45^{{}^{\circ}}}}{2}\sin(\varphi_{\omega_{d}}) \end{array}$$

(10) $$\begin{array}{l} y2_{LPF}=LPF\left\{y(t)\times \sin(\omega_{d}t+45^{{}^{\circ}})\right\}\\ \;=-\frac{A_{\Omega }}{4}\left(\sin(\omega_{\Omega }t+\varphi_{\omega_{d}+\omega_{\Omega }}-45^{{}^{\circ}})+\sin(-\omega_{\Omega }t+\varphi_{\omega_{d}-\omega_{\Omega }}-45^{{}^{\circ}})\right)\\ +\;\frac{A_{q}}{2}\cos(\varphi_{\omega_{d}}-45^{{}^{\circ}})+\frac{A_{45^{{}^{\circ}}}}{2}\cos(\varphi_{\omega_{d}}) \end{array}$$

After that, $y1_{LPF}$ and $y2_{LPF}$ are added to obtain the phase metric $\Delta \varphi_{y}\text{:}$

(11) $$\begin{array}{l} \Delta \varphi_{y}=y1_{LPF}+y2_{LPF}\\ \;=\underset{\mathrm{Coriolis}\;\mathrm{response}}{\underset{︸}{\frac{A_{\Omega }}{4}\left(\cos(\omega_{\Omega }t+\varphi_{\omega_{d}+\omega_{\Omega }}-45^{{}^{\circ}})+\cos(-\omega_{\Omega }t+\varphi_{\omega_{d}-\omega_{\Omega }}-45^{{}^{\circ}})-\sin(\omega_{\Omega }t+\varphi_{\omega_{d}+\omega_{\Omega }}-45^{{}^{\circ}})-\sin(-\omega_{\Omega }t+\varphi_{\omega_{d}-\omega_{\Omega }}-45^{{}^{\circ}})\right)}}\\ \;+\;\underset{\mathrm{Quadrature}\;\mathrm{response}}{\underset{︸}{\frac{A_{q}}{2}\left(\sin(\varphi_{\omega_{d}}-45^{{}^{\circ}})+\cos(\varphi_{\omega_{d}}-45^{{}^{\circ}})\right)}}+\underset{45^{{}^{\circ}}\mathrm{additional}\;\mathrm{force}\;\mathrm{responce}}{\underset{︸}{\frac{A_{45^{{}^{\circ}}}}{2}\left(\cos(\varphi_{\omega_{d}})+\sin(\varphi_{\omega_{d}})\right)}} \end{array}$$

To simplify the analysis, set the input angular rate to a constant value, i.e., $\omega_{\Omega }=0$, Ω(t)=Ω. Then, $\Delta \varphi_{y}$ can be reduced to:

(12) $$\begin{array}{l} \Delta \varphi_{y}\;=\underset{\mathrm{Coriolis}\;\mathrm{response}}{\underset{︸}{\frac{A_{\Omega }}{2}\left(\cos(\varphi_{\omega_{d}}-45^{{}^{\circ}})-\sin(\varphi_{\omega_{d}}-45^{{}^{\circ}})\right)}}+\;\underset{\mathrm{Quadrature}\;\mathrm{response}}{\underset{︸}{\;\frac{A_{q}}{2}\left(\sin(\varphi_{\omega_{d}}-45^{{}^{\circ}})+\cos(\varphi_{\omega_{d}}-45^{{}^{\circ}})\right)}}+\underset{45^{{}^{\circ}}\mathrm{additional}\;\mathrm{force}\;\mathrm{response}}{\underset{︸}{\frac{A_{45^{{}^{\circ}}}}{2}\left(\cos(\varphi_{\omega_{d}})+\sin(\varphi_{\omega_{d}})\right)}}\;\\ \;=\;(\frac{\sqrt{2}A_{\Omega }}{2}+\frac{A_{45^{{}^{\circ}}}}{2})\cos(\varphi_{\omega_{d}})+(\frac{\sqrt{2}A_{q}}{2}+\frac{A_{45^{{}^{\circ}}}}{2})\sin(\varphi_{\omega_{d}}) \end{array}$$

A parameter $k_{45^{{}^{\circ}}}$ is set to indicate the proportional relationship between the 45° additional force response amplitude and the quadrature response amplitude, i.e., $A_{45^{{}^{\circ}}}=k_{45^{{}^{\circ}}}A_{q}$. When setting $k_{45^{{}^{\circ}}}=-\sqrt{2}$, the quadrature response term is canceled by the 45° additional force response term. Then, (12) can be simplified as

(13) $$\Delta \varphi_{y}=\;\frac{\sqrt{2}}{2}\;\left(A_{\Omega }-A_{q}\right)\cos(\varphi_{\omega_{d}})\;$$

It can be seen that, unlike the method of [16], the phase metric $\Delta \varphi_{y}$ in the 45° AFD-RM method only contains a $\cos(\varphi_{\omega_{d}})$ item and has no $\sin(\varphi_{\omega_{d}})$ item. Therefore, it can be used as a basis for judging whether the mode matched.

**Figure 4:**
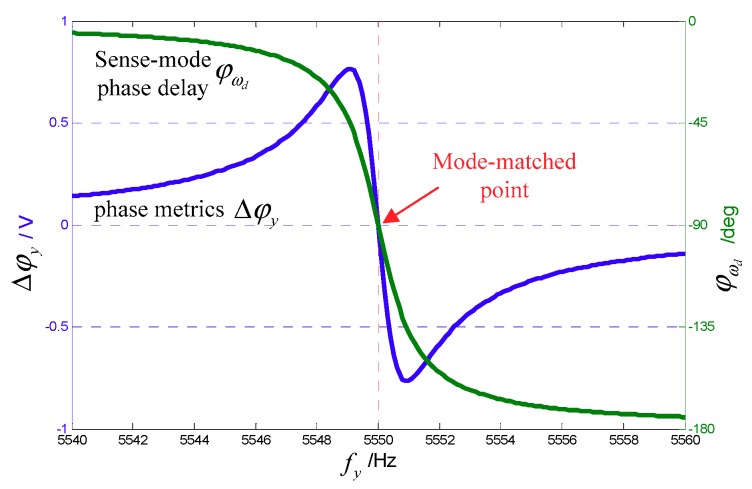
Relationship between mode frequency split and sense phase delay and phase metrics.

Figure 4 shows the relationship curves of phase metrics $\Delta \varphi_{y}$ and sense-mode phase delay $\varphi_{\omega_{d}}$ with the sense-mode frequency $f_{y}$ ($f_{y}=\omega_{y}/2\pi$), where the drive-mode frequency $f_{x}=5550\;\mathrm{Hz}$, sense-mode *Q* value $Q_{y}=5000$, and $A_{q}=-1\times A_{ys}$, $A_{\Omega }=-0.5\times A_{ys}$. We can see that when mode-matched, ($f_{y}=f_{x}=5550\;\mathrm{Hz}$), $\varphi_{\omega_{d}}=-90^{{}^{\circ}}$, and $\Delta \varphi_{y}=0$. When $f_{y}<f_{x}$, $\Delta \varphi_{y}$ is always positive; when $f_{y}>f_{x}$, $\Delta \varphi_{y}$ is always negative. Therefore, we can use $\Delta \varphi_{y}$ as an input variable to control the tuning voltage by the PI controller, and whether $\Delta \varphi_{y}$ is equal to 0 is used as a judgment basis for the mode-matched condition. In addition, in the frequency range near $f_{y}\approx f_{x}$, the $\Delta \varphi_{y}$ value changes quite sensitively, which helps the system to quickly converge and improve the control accuracy.

### 3.3. Influence Analysis of $k_{45^{{}^{\circ}}}$

It is noteworthy that we set $A_{45^{{}^{\circ}}}=k_{45^{{}^{\circ}}}A_{q}$, $k_{45^{{}^{\circ}}}=-\sqrt{2}$. In practical applications, the realization steps of the coefficient $k_{45^{{}^{\circ}}}=-\sqrt{2}$ are as follows: First, the quadrature response amplitude $A_{q}$ of the sense mode is measured when the drive mode is in steady state. Then, a 45° additional force signal $x_{45^{{}^{\circ}}}(t)=A_{45^{{}^{\circ}}}^{\prime }\sin(\omega_{d}t+45^{{}^{\circ}})$ is applied to the sense mode and the response amplitude $A_{45^{{}^{\circ}}}$ is measured. Finally, the $A_{45^{{}^{\circ}}}^{\prime }$ value is reasonably adjusted, and the $A_{45^{{}^{\circ}}}^{\prime }$ value that can satisfy $A_{45^{{}^{\circ}}}=-\sqrt{2}A_{q}$ is selected as the final set value. For each gyroscope, this process is performed only once.

**Figure 5:**
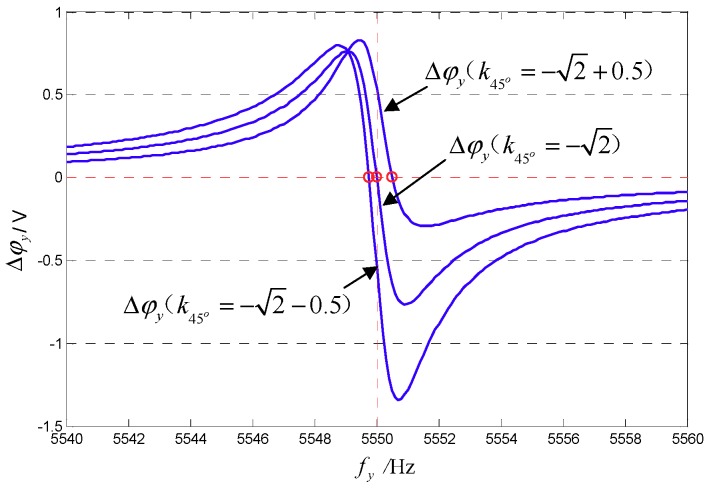
Relationship between $\Delta \varphi_{y}$ and sense-mode frequency when $k_{45^{{}^{\circ}}}$ takes different values.

**Figure 6:**
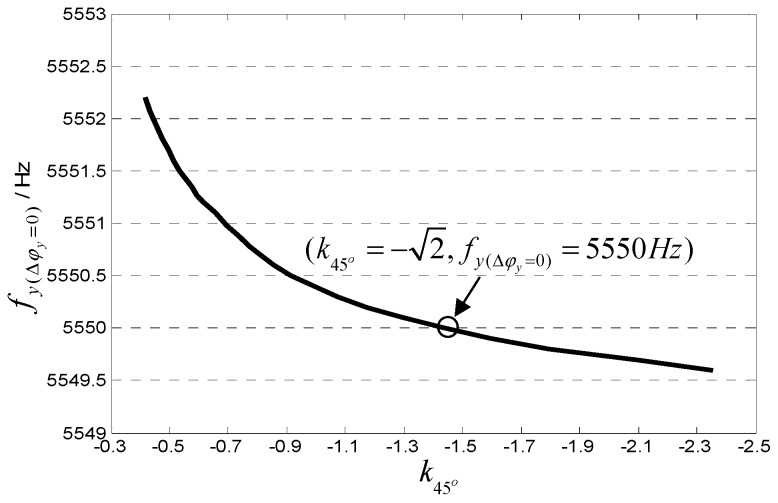
Relationship between $k_{45^{{}^{\circ}}}$ and the frequency $f_{y(\Delta \varphi_{y}=0)}$.

In order to evaluate the influence of the coefficient $k_{45^{{}^{\circ}}}$ value on the accuracy of the 45° AFD-RM control system, the degree of influence of the phase metrics $\Delta \varphi_{y}$ on $k_{45^{{}^{\circ}}}$ is analyzed. Figure 5 shows the relationship between $\Delta \varphi_{y}$ and the sense-mode frequency when $k_{45^{{}^{\circ}}}$ takes different values. It can be seen that when $k_{45^{{}^{\circ}}}\neq -\sqrt{2}$, the sense-mode resonant frequency corresponding to $\Delta \varphi_{y}=0$ ($f_{y(\Delta \varphi_{y}=0)}$) is not 5550 Hz. Figure 6 shows the relationship between $k_{45^{{}^{\circ}}}$ and the frequency $f_{y(\Delta \varphi_{y}=0)}$. It can be seen that the change in coefficient $k_{45^{{}^{\circ}}}$ has little effect on $f_{y(\Delta \varphi_{y}=0)}$. When $k_{45^{{}^{\circ}}}$ doubles up or down, $f_{y(\Delta \varphi_{y}=0)}$ changes within 1 Hz. Therefore, the 45° AFD-RM control system is not very sensitive to the value of $k_{45^{{}^{\circ}}}$. For this reason, it is necessary to properly adjust only the value $A_{45^{{}^{\circ}}}^{\prime }$ so that $A_{45^{{}^{\circ}}}\approx -\sqrt{2}A_{q}$ in practical applications, which is easy to implement.

### 3.4. Angular Rate Detection Output

The traditional phase demodulation method is used to obtain the Coriolis response amplitude (angular rate), the sense-mode output y(t) is multiplied by the reference signal $-\sin(\omega_{d}t)$ and $\cos(\omega_{d}t)$; the Coriolis response amplitude r(t) and quadrature response amplitude q(t) are obtained after passing through the LPF:

(14) $$\left\{ \begin{array}{l} r(t)=\frac{A_{\Omega }}{4}\left(\sin(\omega_{\Omega }t+\varphi_{\omega_{d}+\omega_{\Omega }})+\sin(-\omega_{\Omega }t+\varphi_{\omega_{d}-\omega_{\Omega }})\right)-\frac{A_{q}}{2}\cos(\varphi_{\omega_{d}})-\frac{A_{45^{{}^{\circ}}}}{2}\cos(\varphi_{\omega_{d}}+45^{{}^{\circ}})\\ q(t)=\frac{A_{\Omega }}{4}\left(\cos(\omega_{\Omega }t+\varphi_{\omega_{d}+\omega_{\Omega }})+\cos(-\omega_{\Omega }t+\varphi_{\omega_{d}-\omega_{\Omega }})\right)+\frac{A_{q}}{2}\sin(\varphi_{\omega_{d}})+\frac{A_{45^{{}^{\circ}}}}{2}\sin(\varphi_{\omega_{d}}+45^{{}^{\circ}}) \end{array}\right.\;$$

For $\varphi_{\omega_{d}+\omega_{\Omega }}$ and $\varphi_{\omega_{d}-\omega_{\Omega }}$, because it is the phase delay caused by the signal $\cos\left((\omega_{d}+\omega_{\Omega })t\right)$ and $\cos\left((\omega_{d}-\omega_{\Omega })t\right)$ through the sense mode, according to the phase-frequency characteristics at resonance state, $\varphi_{\omega_{d}+\omega_{\Omega }}$ and $\varphi_{\omega_{d}-\omega_{\Omega }}$ are symmetrical about $-90^{{}^{\circ}}$ in mode-matching, i.e., $\varphi_{\omega_{d}-\omega_{\Omega }}+\varphi_{\omega_{d}+\omega_{\Omega }}=\;-180^{{}^{\circ}}$. Therefore, when $A_{45^{{}^{\circ}}}=-\sqrt{2}A_{q}$ and is mode-matched, Equation (14) can be simplified as:

(15) $$\left\{ \begin{array}{l} r(t)=\frac{A_{q}}{2}+\frac{A_{\Omega }}{2}\sin(\omega_{\Omega }t+\varphi_{\omega_{d}+\omega_{\Omega }})\\ q(t)=\frac{A_{\Omega }}{2}\cos(\omega_{\Omega }t+\varphi_{\omega_{d}+\omega_{\Omega }}) \end{array}\right.$$

It can be seen that the Coriolis response amplitude r(t) contains not only the Coriolis response but also a relatively constant quadrature response term $\frac{A_{q}}{2}$. When there is no angular rate input (i.e., $A_{\Omega }=0,\;\omega_{\Omega }=0$), then $r(t)=\frac{A_{q}}{2}$, which is the zero-bias output; also, q(t) is 0, because the additional force cancels the quadrature force. In this study, q(t) was used to estimate the actual mode frequency split in mode-matched condition.

Because the gyroscope cannot be swept to determine the mode frequency during normal work, the frequency split can only be estimated by r(t) and q(t). When there is no angular rate input and $A_{45^{{}^{\circ}}}=-\sqrt{2}A_{q}$, Equation (14) can be expressed as

(16) $$\left\{ \begin{array}{l} r(t)=-\frac{A_{q}}{2}\sin(\varphi_{\omega_{d}})\\ q(t)=-\frac{A_{q}}{2}\cos(\varphi_{\omega_{d}}) \end{array}\right.\;$$

The actual phase delay can be estimated by φωd=arctan(r(t)q(t)). Then, on the basis of the phase-frequency curve obtained by the frequency sweep in advance, the actual mode frequency split in mode-matched state can be estimated.

## 4. Simulation Analysis

**Figure 7:**
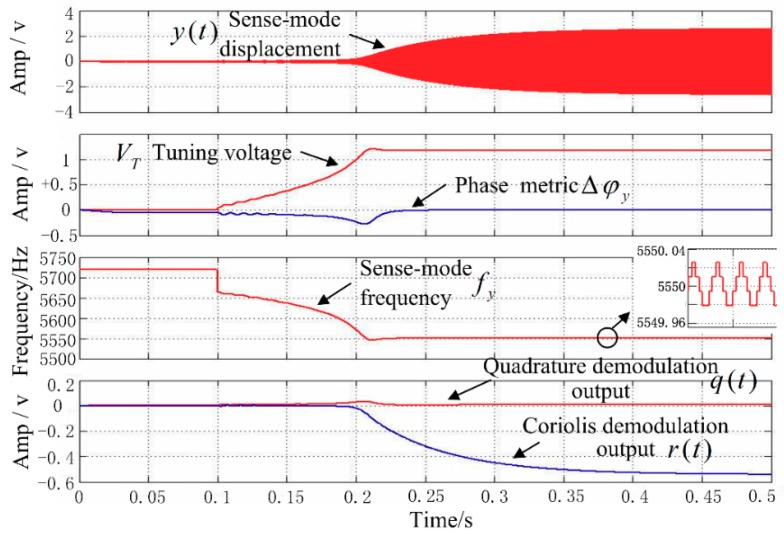
Mode-matching process.

Simulation analysis was performed in MATLAB Simulink; the simulation parameters were set according to the existing MW-AVG silicon micromachined wheel vibratory gyroscope in the research group. The gyroscope parameters are shown in Table 1. In addition, the tuning voltage bias $V_{ref}$ = 2.5 V and the initial input Coriolis force was zero. After the drive-mode closed-loop control became stable, the mode-matching process was started at *t* = 0.1 s. The waveform of the matching process is shown in Figure 7. It can be seen that after starting the mode-matching, because $f_{y}>f_{x}$, the phase metric $\Delta \varphi_{y}<0$, so that the tuning voltage $V_{T}$ continuously rises to 1.21 V, finally making $\Delta \varphi_{y}=0$ and stabilizing in the mode-matched state. At this time, the sense-mode displacement amplitude y(t) reaches a maximum, the sense-mode frequency $f_{y}$ decreases from 5719 Hz to 5550 Hz, and the mode frequency split is controlled within 0.03 Hz.

**Figure 8:**
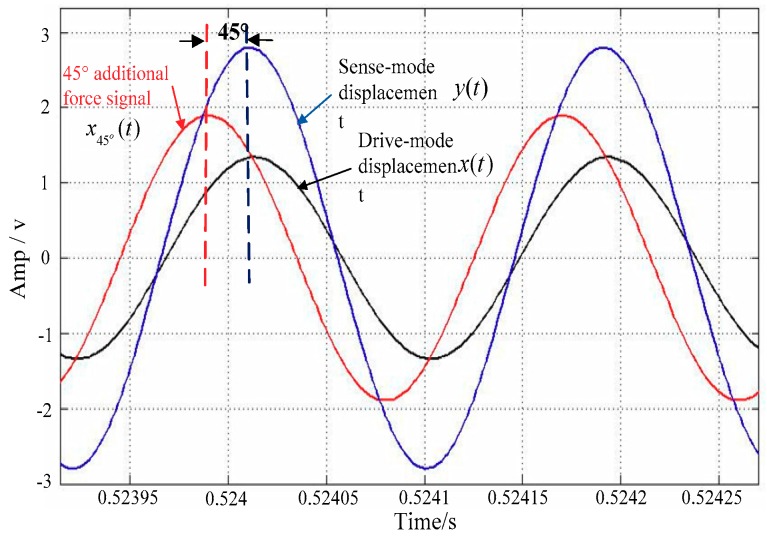
Phase relationship of some signals in mode-matched state.

In theoretical calculations, when $A_{\Omega }=0$ and $A_{45^{{}^{\circ}}}=-\sqrt{2}A_{q}$, the signal phase relationship in mode-matched state is $x_{45^{{}^{\circ}}}(t)=A_{45^{{}^{\circ}}}^{\prime }\sin(\omega_{d}t+45^{{}^{\circ}})$, $x(t)=A_{x}\sin(\omega_{d}t)$, and $y(t)=\frac{\sqrt{2}}{2}\;A_{q}\sin(\omega_{d}t)$. Figure 8 shows the phase diagram of these signals in mode-matched state. It can be seen that without the input of Coriolis force, the sense-mode displacement y(t) is in phase with the drive-mode displacement x(t) and is 45° delayed from the 45° additional force signal $x_{45^{{}^{\circ}}}(t)$. This is exactly the same as the theoretical calculation, which proves the correctness of the proposed scheme.

## 5. Experimental Analysis

### 5.1. Experimental Setup

**Figure 9:**
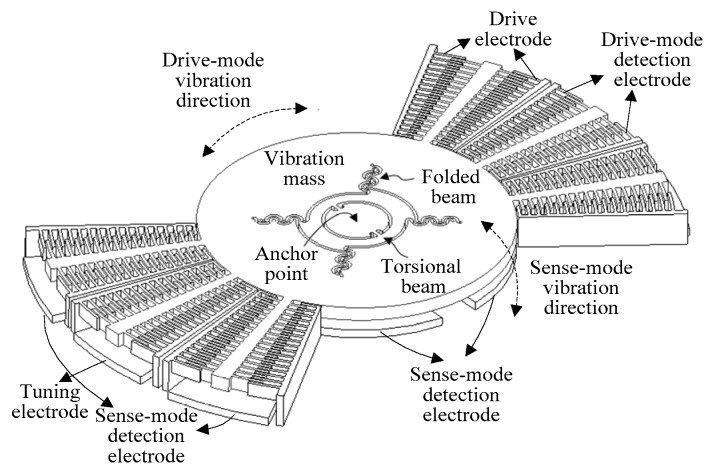
Structure diagram of wheel vibratory gyroscope.

**Figure 10:**
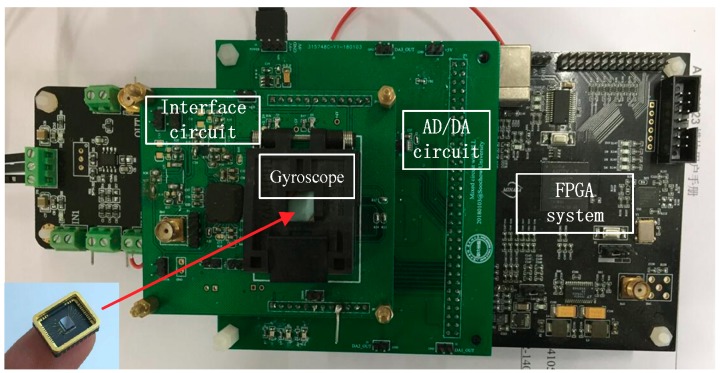
Control circuit of wheel vibratory gyroscope.

Taking an MW-AVG silicon micromachined wheel vibratory gyroscope as the experimental object, a gyroscope drive-mode closed-loop and mode-matching control system was realized by Field Programmable Gate Array (FPGA). The relevant data were collected through the serial port and LabVIEW software, with a sampling rate of 180 Hz. Table 1 shows some electrical parameters of the gyroscope. The gyroscope internal structure and the control circuit are shown in Figure 9 and Figure 10. In addition, the tuning voltage bias $V_{ref}$ = 2.5 V, i.e., the voltage applied to the tuning electrode was $V_{p}$ = $V_{T}$ + $V_{ref}$.

There were two pairs of plate electrodes in the gyroscope sense mode; one pair was used as sense detection electrodes, one of the other pair was used as a tuning electrode, and the other was used to apply the 45° additional force signal $x_{45^{{}^{\circ}}}(t)$. In addition, the drive-mode control system used the traditional PLL + AGC method [22,23], where the reference amplitude of the drive-mode displacement x(t) was set to 0.05 V. The Coriolis response detection method for the sense mode used open-loop detection. After relevant measurements, the $A_{45^{{}^{\circ}}}^{\prime }$ value was set to 0.1 V, so that $A_{45^{{}^{\circ}}}\approx -\sqrt{2}A_{q}$. 

### 5.2. Mode-Matching Process

**Figure 11:**
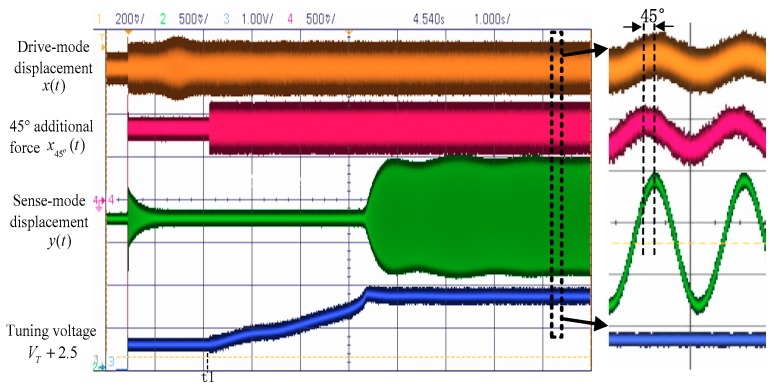
Waveforms in the mode-matching process.

In the experiment, the mode-matching process was started at *t* = 2 s. The waveform of the startup process is shown in Figure 11. It can be seen that when mode-matching is started, the tuning voltage ($V_{p}=V_{T}+V_{ref}$) continuously rises and stabilizes at approximately 3.61 V, and the vibration displacement of the sense mode rapidly increases. The right side of Figure 11 shows that when the matching is stable, the drive-mode displacement x(t) is in phase with the sense-mode displacement y(t) and is 45° delayed from the 45° additional force signal $x_{45^{{}^{\circ}}}(t)$, which is exactly the same as the simulation result and shows that mode-matching has been achieved.

**Figure 12:**
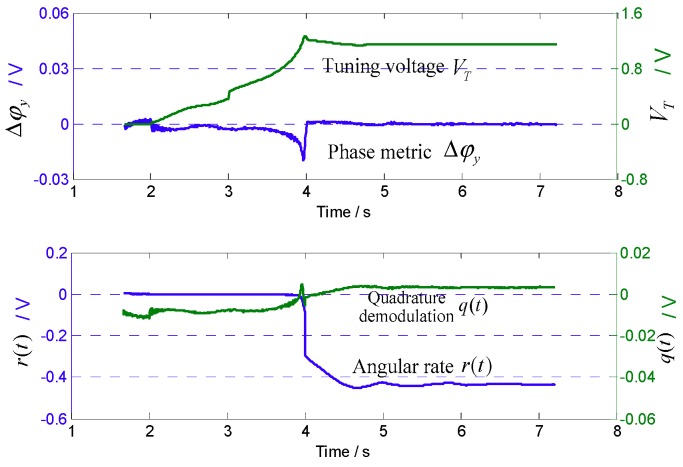
Related variable curves in the mode-matching process.

Figure 12 shows variation of the relevant signal during the start of matching. It can be seen that at the beginning, because $f_{y}>f_{x}$, the phase metric $\Delta \varphi_{y}<0$, so that the tuning voltage $V_{T}$ rises. In the close mode matching, $V_{T}$ rises sharply and then quickly converges to 0, which is consistent with the trajectory shown in Figure 4. The condition $\Delta \varphi_{y}=0$ indicates mode matching. At this time, $V_{T}$ stabilizes to approximately 1.11 V. In addition, it can be seen that the Coriolis demodulation result r(t) stabilizes to a negative value, $\frac{A_{q}}{2}$, which is the zero-bias output of the angular rate detection.

According to Equation (17), the actual mode frequency split under 45° AFD-RM real-time matching is estimated by r(t) and q(t). In the steady state shown in Figure 12, r(t)=−0.4366   V and q(t)=0.0083   V, so the phase delay $\varphi_{\omega_{d}}=-88.91{}^{\circ}$. Then, according to the measured sense-mode phase-frequency curve, the mode frequency split is within approximately 0.1 Hz.

### 5.3. Gyroscope Performance Analysis

In the experiment, the gyroscope performance under real-time mode-matched (45° AFD-RM), one-time manual mode-matched (OMM), and mismatched conditions was compared and analyzed. For the mismatched condition, two fixed tuning voltage were set: (1) $V_{p}=3V$, corresponding to a frequency split Δf of approximately 60 Hz; and (2) $V_{p}=3.5V$, corresponding to a frequency split Δf of approximately 10 Hz. 

For the OMM condition, this was achieved by manually adjusting the tuning voltage. However, because the resonant frequency of the gyroscope changes with the environmental parameters, the matching voltages at different times may be different. In addition, around the mode-matched point, the gain $A_{ys}$ of sense mode is greatly affected by the frequency split. Therefore, for high-Q gyroscopes, OMM cannot achieve perfect mode matching, and only approximate matching can be achieved. By performing manual fine adjustment at normal temperature, the mode matching was optimal at the tuning voltage $V_{p}=3.652V$. At this time, in the case of no angular rate input, the demodulation outputs of the sense mode were q(t)=−0.098   V, r(t)=0.067   V. Then, the phase delay was calculated as $\varphi_{\omega_{d}}=-55.64{}^{\circ}$ and the mode frequency split was estimated to be approximately 1 Hz.

In order to test the scale factor, the Coriolis force must be applied to the sense mode. In this study, the virtual Coriolis force calibration method [24] was used to apply the virtual Coriolis force signal $F_{\Omega }(t)=A_{\Omega }^{\prime }\cos\left(\omega_{\Omega }t\right)\cos(\omega_{d}t)$ on the sense-mode electrodes through the FPGA, and the corresponding actual angular rate was calibrated by the rate table. In experiments, the input virtual Coriolis force was set to a constant value, $\omega_{\Omega }=0$.

**Figure 13:**
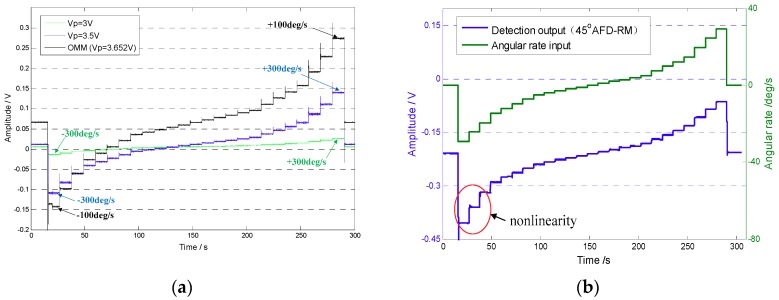
Detection output at different angular rate inputs: (**a**) mismatched and one-time manual mode-matched (OMM) conditions; (**b**) 45° AFD-RM condition (mode-matched).

First, under the normal temperature environment and using the open-loop detection method, the gyroscope under fixed tuning voltage and real-time mode-matching conditions was tested for scale factor. For the fixed tuning voltage condition, the range of amplitude $A_{\Omega }^{\prime }$ was −0.6 V to 0.6 V, corresponding to an actual angular rate of approximately −300°/s to 300°/s, which was divided into 24 levels. For the 45° AFD-RM real-time mode-matching and OMM conditions, because the linear measurable range is smaller when mode-matched, the amplitudes $A_{\Omega }^{\prime }$ were set to −0.06 V to 0.06 V and −0.2 V to 0.2 V, respectively, and the corresponding actual angular rates were approximately −30°/s to 30°/s and −100°/s to 100°/s, respectively. The gyroscope detection results at different input angular rates are shown in Figure 13. 

It can be seen that under open-loop detection, the Coriolis response becomes larger as the mode frequency split Δf decreases. At −300°/s to 300°/s, the Coriolis response amplitude changes approximately 0.04 V at $V_{p}=3V$ and approximately 0.25 V at $V_{p}=3.5V$. In mode-matched condition, the amplitude change reaches 0.35 V at −30°/s to 30°/s, which means that the sensitivity is increased by several dozen times. However, as can be seen from Figure 13b, nonlinearity occurs when the response is large, which is the reason why the measurable range under the mode-matched condition is small.

**Figure 14:**
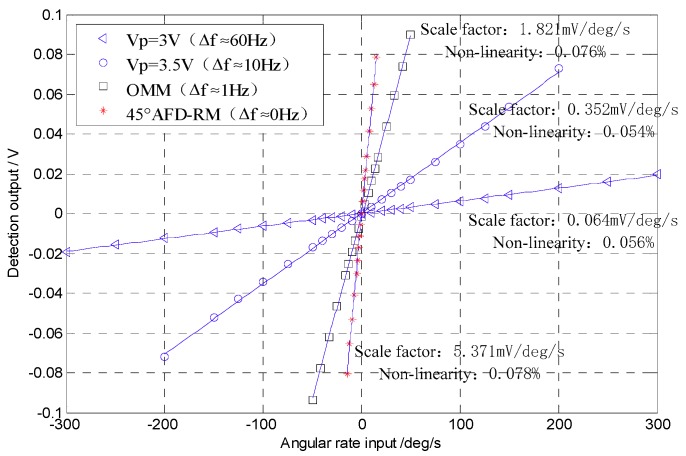
Scale factor.

**Figure 15:**
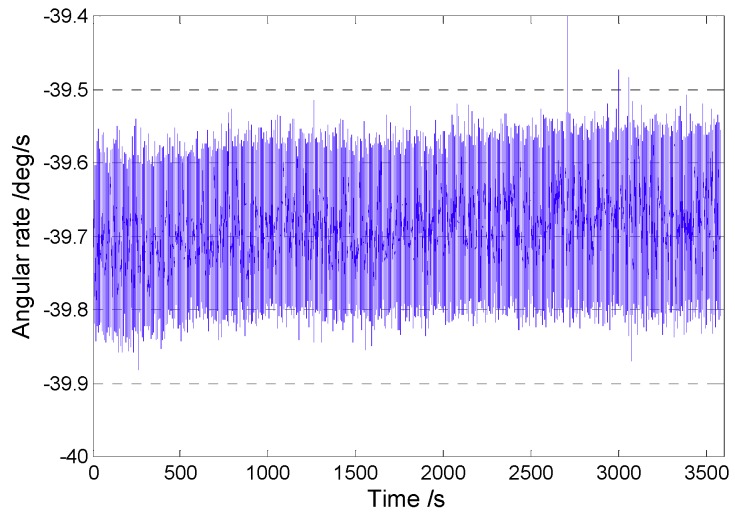
Zero-bias output under 45° AFD-RM.

**Figure 16:**
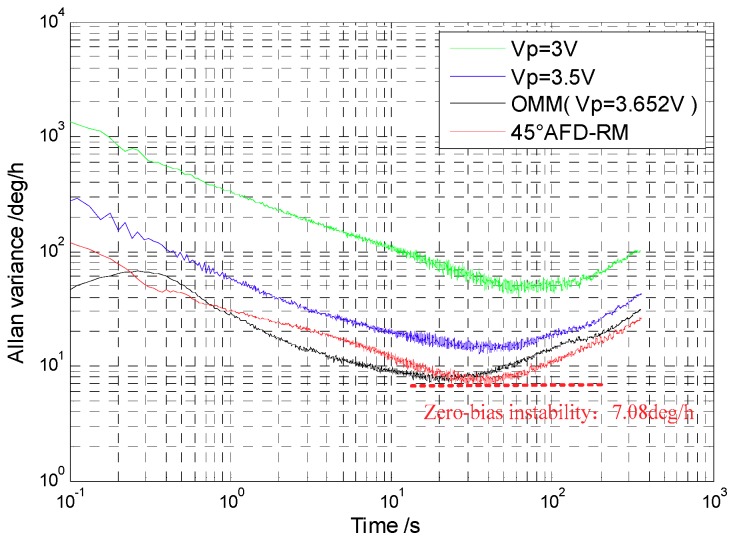
Allan variance curve.

The Coriolis response test results under the four conditions above were counted. With the zero-bias output normalized to zero, the data in an angular rate range with good linearity were selected to perform linear fitting, so that a scaling factor was obtained, as shown in Figure 14. In addition, after the gyroscope worked stably for 30 min, the gyroscope zero-bias output was collected for 1 h and the zero-bias performance was analyzed by the Allan variance [25]. The zero-bias output under 45° AFD-RM real-time mode-matching is shown in Figure 15, and the Allan variance curve under the four conditions is shown in Figure 16. The results of scale factor, nonlinearity, zero-bias instability, and angle random walk are shown in Table 2.

For the mode-matched and mismatched conditions, it can be seen that under the 45° AFD-RM real-time mode-matched condition, the scale factor reaches 5.371 mV/°/s, which is 84 times that of $V_{p}=3V$ and 15 times that of $V_{p}=3.5V$. This is because the sense-mode gain is the largest and the mechanical sensitivity is the highest under the mode-matched condition. In addition, under mode-matching, the phase delay in the sense mode is constant, which contributes to the accuracy of Coriolis demodulation, so zero-bias instability and angle random walk performance also improved compared to the mismatched conditions, reaching 7.08°/h and 0.367°/$\sqrt{h}$, respectively, which is 46.4% and 45.5% lower than $V_{p}=3.5V$. However, the measurable range is ±20°/s, which decreased significantly, because under the mode-matched condition, the gyroscope sense-mode vibration displacement (Coriolis response) is the largest under the same angular rate input; due to the limitations of the gyroscope structure and the detection circuit, the measurable range is reduced. However, this problem can be mitigated by the force feedback closed-loop detection method.

For the OMM condition, there is a frequency split of approximately 1 Hz at normal temperature. Therefore, the scale factor of 45° AFD-RM is 2.9 times that of OMM. However, its zero-bias instability and angle random walk are approximately equal to OMM. This is because the 45° AFD-RM introduces an additional force and a tuning voltage closed-loop control system for real-time matching, which results in additional noise. However, this problem can also be mitigated by force feedback closed-loop detection. In other words, the quadrature force feedback is used to correct the quadrature force, so as to reduce the value of $A_{q}$, thereby reducing the amplitude $A_{45^{{}^{\circ}}}$ of 45° additional force ($A_{45^{{}^{\circ}}}=-\sqrt{2}A_{q}$) and the electrical coupling noise.

It is worth noting that in the actual applications, the biggest drawback of OMM is that it uses fixed voltage tuning, which causes the mode-matching to be affected by environmental parameters (especially temperature), which will result in poor accuracy and reliability of angular rate detection. In this paper, the 45°AFD-RM can adjust the matching voltage in real time, so that the gyroscope maintains the mode-matched state, which makes it robust to environmental changes. 

### 5.4. Temperature Experiment

The temperature of the chamber was set to 30 °C, 50 °C, and 70 °C and OMM and 45° AFD-RM were performed. Among them, OMM is manually matched and fixed tuning voltage $V_{p}=\;3.652V$ at normal temperature.

**Figure 17:**
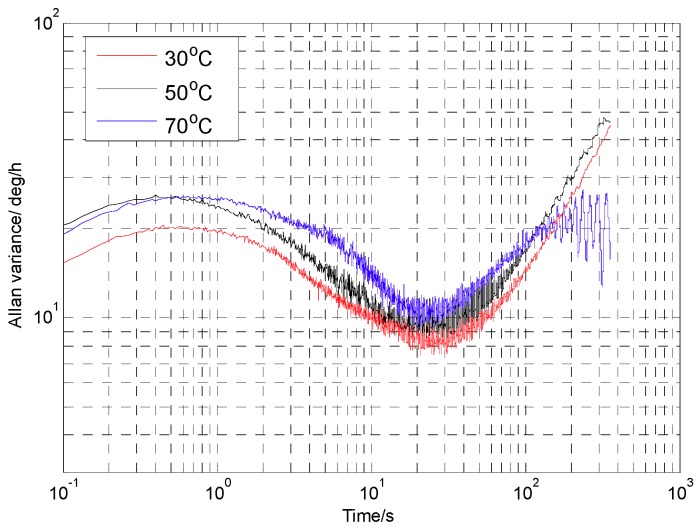
Allan variance curve at different temperatures.

Zero-bias Allan variance graphs of 45° AFD-RM under three different temperatures are shown in Figure 17. The higher the temperature, the worse the bias instability, because as temperature rises, the *Q* value will decrease. Both of these changes will induce a loss of mechanical sensitivity and increase the noise. 

The tuning voltage and performance parameters are calculated and summarized in Table 3. It can be seen that the tuning voltage of the 45° AFD-RM changes with temperature, keeping the gyroscope in the mode-matched state. In addition, with the change of temperature, 45° AFD-RM is better than OMM in terms of zero-bias instability and angular random walk. This is because the frequency split of the gyroscope changes with temperature, and the fixed tuning voltage in the OMM cannot achieve mode-matching, which further reduces the sensitivity of the gyroscope.

## 6. Conclusions

This paper presents an automatic real-time mode-matching method called 45° AFD-RM. The difference from the existing external force-based real-time mode-matching method is that 45° phase-shifted additional force and 45° phase-shifted demodulation are used, so as to obtain a phase metric that has no interference by Coriolis force and quadrature force, thereby reducing the limitations of method implementation and improving matching accuracy. The experimental results show that the 45° AFD-RM method can accurately achieve mode-matching, significantly increase the scale factor, and reduce bias instability and angle random walk.

However, because the open-loop detection method was adopted in this study, the measurable range is small and the improvement of zero-bias instability and angle random walk is not ideal. These problems can be solved by closed-loop detection. However, because the Coriolis demodulation output is different under the 45° AFD-RM and fixed tuning voltage method, the traditional force feedback closed-loop detection method cannot be used directly. Therefore, in future research, a forced feedback closed-loop detection method that matches the 45° AFD-RM will be constructed to further improve gyroscope performance.

## Figures and Tables

**Table 1:** Main electrical parameters of the gyroscope.

Parameter	Drive mode	Sense mode
Inertia *I_x_*, *I_y_* (kg·m^2^)	1.11 × 10^−14^	8.86 × 10^−15^
Resonance frequency *f_x_*, *f_y_* (Hz)	5550	5719
Quality value *Q_x_*, *Q_y_*	2.2 × 10^5^	5200
Conversion factor of voltage to torque *k_vtx_*, *k_vty_* (N/V)	5.28 × 10^−11^	4.18 × 10^−9^
Conversion factor of swing angle to capacitance *k_xc_*, *k_yc_* (F/rad)	6.3 × 10^−12^	446 × 10^−12^
Conversion factor of detector capacitance to voltage *k_cvx_*, *k_cvy_* (V/F)	1.3 × 10^12^	10.4 × 10^12^
Coupling stiffness *k_yx_* (N/m)	—	2.2 × 10^−9^

**Table 2:** Performance test results (normal temperature, open-loop detection).

Matching Condition	Performance Parameters				
	Scale Factor (mV/°/s)	Nonlinearity (%)	Measurable Range (°/s)	Zero-Bias Instability (°/h)	Angle Random Walk (°/$\sqrt{h}$)
$V_{p}=3V$(Δf ≈ 60 Hz)	0.064	0.056	Greater than ±300	42.65	3.671
$V_{p}=3.5V$(Δf ≈ 10 Hz)	0.352	0.054	±200	13.23	0.667
OMM (Δ*f* ≈ 1 Hz)	1.821	0.076	±50	7.52	0.325
45° AFD-RM (Δ*f* ≈ 0 Hz)	5.371	0.078	±20	7.08	0.367

**Table 3:** Gyroscope performance at different temperatures (open-loop detection).

Temperature (°C)	45° AFD-RM			OMM		
	Tuning Voltage (V)	Zero-Bias Instability (°/h)	Angle Random Walk (°/$\sqrt{h}$)	Tuning Voltage (V)	Zero-Bias Instability (°/h)	Angle Random Walk (°/$\sqrt{h}$)
30	3.6372	7.76	0.328	3.652	8.26	0.351
50	3.6253	8.84	0.412	3.652	10.03	0.482
70	3.6185	9.59	0.465	3.652	11.32	0.616
